# Effect of preparation type on the accuracy of different intraoral scanners: An in vitro study at different levels of accuracy evaluation

**DOI:** 10.1111/jerd.12949

**Published:** 2022-08-02

**Authors:** Jaafar Abduo, David Laskey

**Affiliations:** ^1^ Melbourne Dental School Melbourne University Melbourne Victoria Australia

**Keywords:** crown, inlay, margin, onlay, precision, trueness

## Abstract

**Objective:**

Evaluation of the effect of preparation type (inlay, onlay, and crown) on the accuracy of different intraoral scanning (IOS) systems at the preparation and arch segment levels.

**Materials and Methods:**

Three molars were prepared for inlay, onlay, and crown. Each preparation was scanned 10 times by CEREC Omnicam, Trios 3 (TS), and Medit i500 scanners. Each image was trimmed twice. The first trimming produced a preparation image (PI), and the second trimming extracted a segment image (SI) that involved the preparation with the adjacent teeth. Trueness and precision were calculated at the PI and SI levels.

**Results:**

At the PI level, all IOS systems had similar trueness pattern for all preparations, where the inlay had the best trueness followed by the crown and onlay. At the SI level, the different preparations showed similar trueness. The precision did not show a clear pattern of superiority for any preparation. The TS was significantly more precise than other IOS systems at the PI and SI levels, for every preparation. The proximal areas suffered from the greatest errors, regardless of preparation type.

**Conclusions:**

The preparation type influenced PI trueness, and the IOS system affected PI and SI precisions.

**Clinical Significance:**

The smaller and less complex preparations have greater IOS accuracy than larger and more complex preparations. As the proximal areas are more affected regardless of the preparation, a more accessible proximal area for scanning is desirable.

## 1 INTRODUCTION

Intraoral scanning (IOS) for the fabrication of indirect restoration has gained considerable popularity over the last few decades. This is largely assisted by the rapid and consistent improvements in the IOS systems. Today, IOS systems have the advantages of ease of use, ergonomic handling, quick scanning, and reliability. Several studies have confirmed the accuracy of IOS systems for multiple applications.^1^, ^2^, ^3^, ^4^, ^5^, ^6^, ^7^, ^8^ However, despite the abundance of studies on the accuracy of IOS systems, there is considerable disparity in their designs.^9^, ^10^, ^11^, ^12^ The variations can be related to the scanning span, the number of units, scanned substrate material and design, and the condition of the scanned teeth.^10^ One of the features that have not been fully investigated is the implication of tooth preparation type on the accuracy of IOS.^1^, ^2^, ^3^, ^9^, ^13^, ^14^, ^15^ Variations in preparation types and designs, such as crown, onlay, and inlay, may interfere with the scanning process and the capturing of the preparation surface details.^16^, ^17^, ^18^, ^19^, ^20^, ^21^ While the preparation type is clinically determined, each preparation exhibits different features that may influence the accuracy of IOS. This involves preparation margins, cavity depth, preparation length, the complexity of preparation geometry, and the level of convergence.^1^, ^2^, ^3^, ^9^, ^13^, ^22^, ^23^ Therefore, IOS‐related recommendations are needed for the different types of preparations.

In addition, a unique limitation of the existing literature on IOS accuracy is the lack of differentiation between the accuracy of the prepared tooth and the accuracy of the scanned segment that involves unprepared adjacent teeth. The preparation level accuracy is certainly most critical for the success of indirect restoration in terms of marginal integrity, cementation thickness, and resistance to caries and periodontal complications.^1^, ^4^, ^24^ However, the segment accuracy is necessary to ensure acceptable seating of the restoration without interfering with the proximal surfaces of adjacent teeth, restoration of contour and emergence profile that mimic the adjacent teeth contours, and re‐establishment of occlusion that is confirmative to the segment. Evaluation at the segment level is likely to reveal different challenges to IOS than preparation level accuracy. Specifically, some preparations along with the adjacent teeth may be easier to record digitally than other preparations. The present study implemented a combined method for IOS accuracy evaluation to determine the effect of different preparation types on the accuracy of IOS. The combined method included the evaluation of the preparation accuracy and segment accuracy by quantifying trueness and precision as per the criteria of International Organization for Standardization (5725‐4:2020).^25^ Therefore, the aim of this study was to evaluate the effect of preparation type (crown, onlay, and inlay) on the accuracy (trueness and precision) of three different IOS systems at the preparation and segment levels. The null hypotheses were that there is no effect of the different preparation types on the accuracy of IOS, and there is no difference between the different IOS systems.

## 2 MATERIALS AND METHODS

**FIGURE 1:**
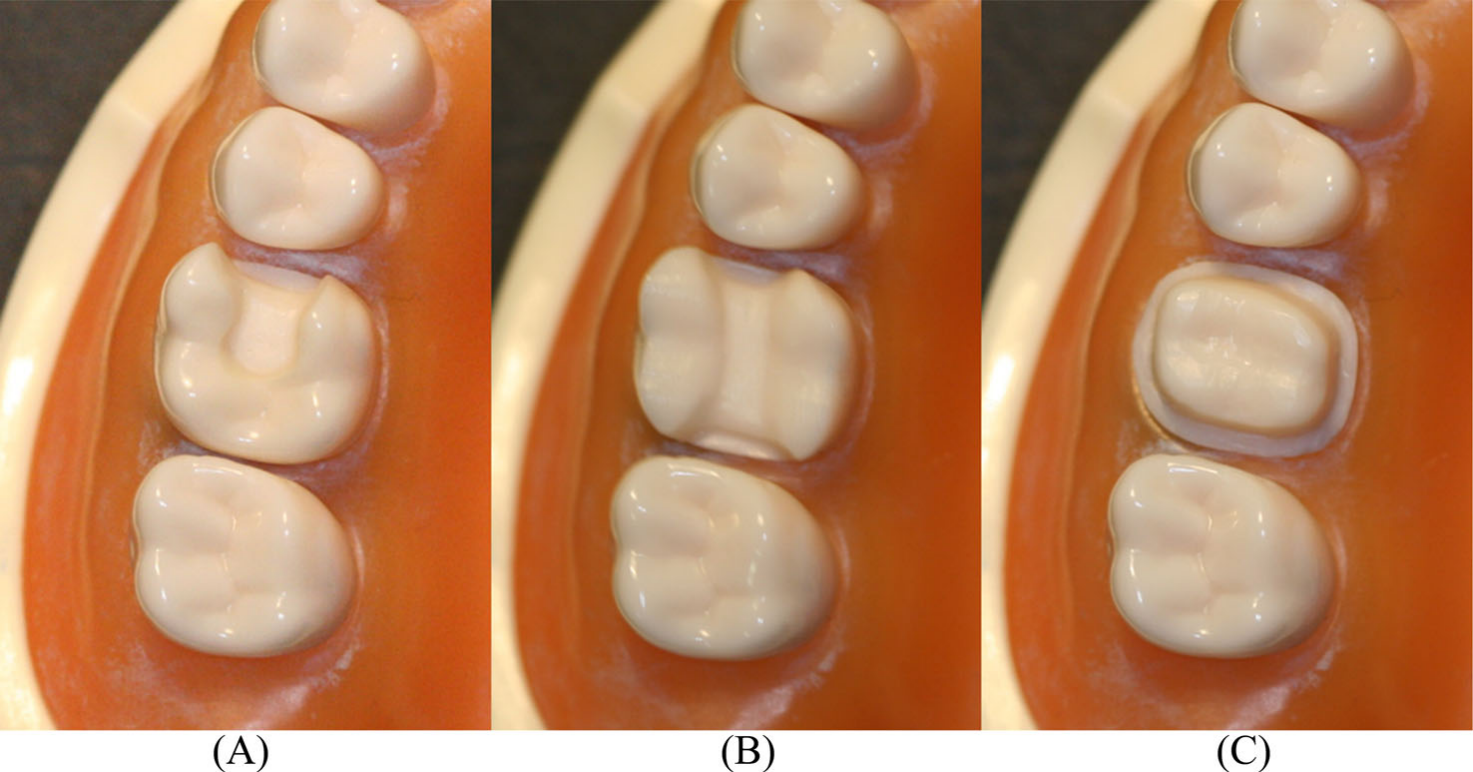
Images illustrating the different preparations. (A) Inlay. (B) Onlay. (C) Crown

On a training model (Nissin Dental Products Inc., Kyoto, Japan), three maxillary right first molars were prepared for inlay, onlay, and crown (Figure 1). The preparations were designed to facilitate IOS and restoration fabrication via computer‐aided design and computer‐aided manufacturing.^16^ For the crown preparation, a 1 mm circumferential rounded shoulder margin was prepared 1 mm supragingivally. Occlusal reduction of 2 mm, 10–15° taper and rounded line angles were incorporated. The inlay preparation mesio‐occlusal outline extended approximately two‐thirds of the occlusal surface and had an occlusal reduction of 2 mm. The mesial box had a 1 mm width and was located 1 mm supragingivally. For the onlay preparation, the cusps were reduced by 2 mm parallel to the occlusal anatomy and the central isthmus was reduced by 3 mm. The mesial and distal boxes of the onlay preparation had a similar design to the mesial box of the inlay preparation. The internal walls of the inlay and onlay preparations had a divergence of 10–15° and all the internal line angles were rounded.

Three intraoral scanners were used in the study, CEREC Omnicam (OC), Medit i500 (MT) and Trios 3 (TS) (Table 1). The manufacturers' instructions on recommended scanning protocol were followed. Each preparation was scanned while it was attached to the training model with intact teeth. The preparations were scanned along with the adjacent teeth of the quadrant and the associated soft tissue. The scanning aimed to achieve continuous and intact virtual surfaces. If the surface was not continuous, the scanning procedure was repeated. All the scanning units were calibrated prior to scanning. Each preparation was scanned 10 times by every scanner. This sample size was confirmed by G*Power software (version 3.1.9.2; University of Dusseldorf, Dusseldorf, Germany). Based on the reported mean difference in the accuracy among the different IOS systems that can be up to 50% with a standard deviation (SD) of 25%,^1^, ^13^, ^24^ an assumption of 80% statistical power and 5% level of significance indicated that at least eight scans were required for each system. After the scanning, all the images were exported into STL files for subsequent analysis.

**TABLE 1:** The intraoral scanners used in this study

System	Manufacturer	Scanning mechanism	Software version
Cerec Omnicam (OC)	Dentsply Sirona, Bensheim, Germany	Video‐type scanning via continuous triangulation	4.6
Medit i500 (MT)	Medit Corp, Seoul, South Korea	Video‐type scanning via continuous triangulation	2.3.1
Trios 3 (TS)	3 Shape, Copenhagen, Denmark	Video‐type scanning via confocal microscopy	21.4

**FIGURE 2:**
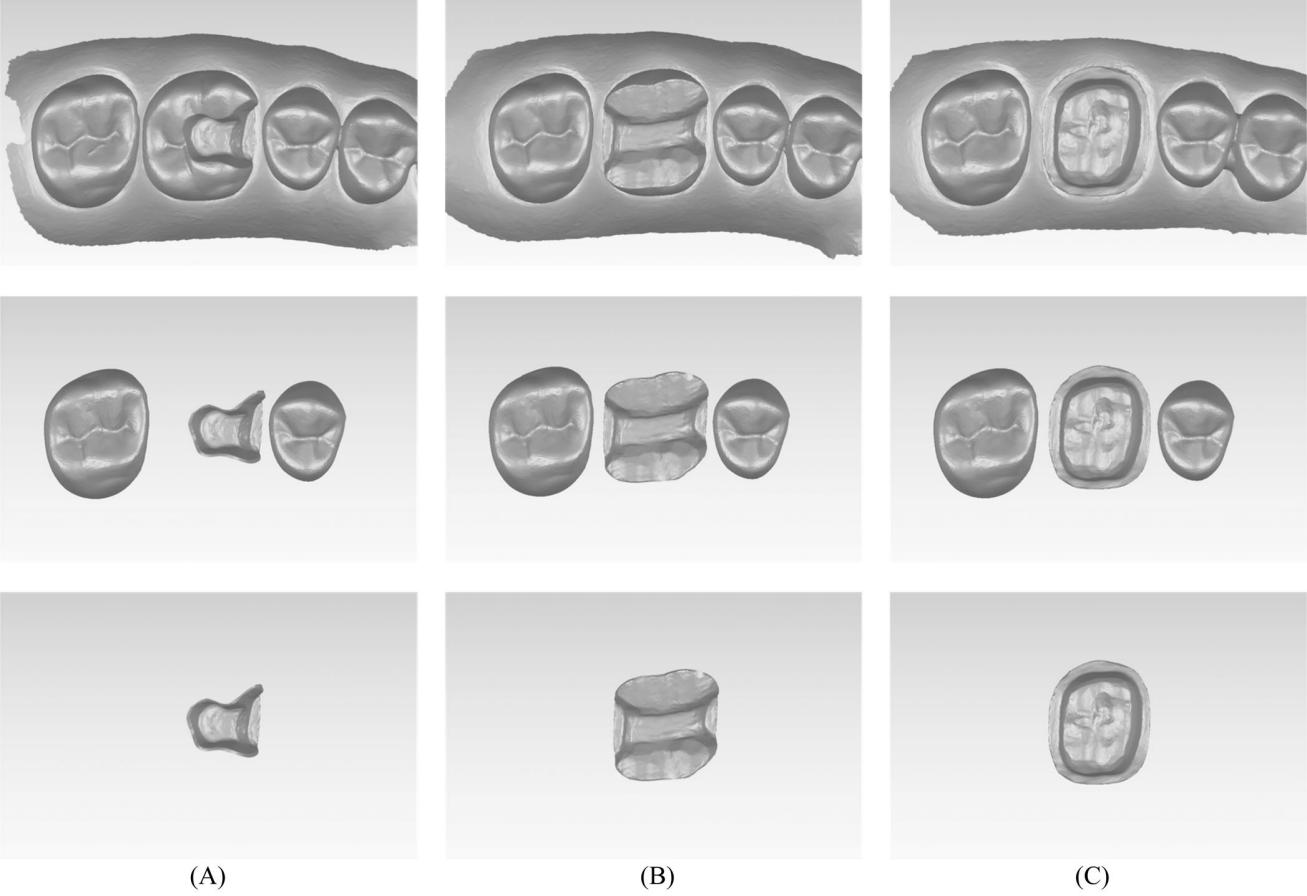
The virtual trimmings of the different preparations. At the middle, the SI that involves the preparation surface and the adjacent teeth. At the bottom, the PI that only includes the preparation surface. (A) Inlay. (B) Onlay. (C) Crown

The STL images were exported to 3D rendering software (Meshmixer, Autodesk Research, Mill Valley, CA, USA) for trimming the excess scanned surfaces. Each image was duplicated to receive two forms of virtual trimming (Figure 2). For the first trimming, the prepared surface just beyond the margin was extracted. This preparation image (PI) was used to evaluate the accuracy of the scanned preparation. The second trimming involved extracting the prepared tooth along with the second premolar and second molar. The segment image (SI) was used to evaluate the accuracy of the prepared tooth along with the adjacent teeth that may influence the seating of the final restoration.

The master virtual images were generated by scanning each prepared tooth separately and in the model with the remaining teeth by a laboratory scanner (Identica T300, Medit Identica, DT Technologies, Davenport, IA, USA). The dual scanning was conducted to ensure all the surfaces of the preparation and adjacent teeth were captured by the laboratory scanner. By using the two trimmed images of every IOS, the accuracies were evaluated at the PI and SI levels. For each level, trueness and precision were calculated as per ISO Standard 5725‐4:2020.^25^ Two forms of accuracy were evaluated for the PI and SI levels, trueness and precision. Trueness is the similarity between the IOS image and the original virtual image (*n* = 10), while precision is the similarity between the different IOS images of the same scanning unit (*n* = 45). Trueness provides information on the deviations of each preparation by every scanning unit, whereas precision measures the repeatability of the scanning unit. The trueness and precision were quantified by the superimposition of virtual images via 3D measurement software (CloudCompare, EDF R&D, Paris, France). The superimposition was performed initially by marking four identical well‐distributed points on the two images for point‐to‐point alignment. The points were located at the corners of each preparation. For the inlay preparation, the points were the four corners of the preparation floor, while for onlay and crown preparations, cusp tips of the mesiobuccal, distobuccal, mesiopalatal, and distopalatal cusps were selected. For the SI superimposition, the most mesial, distal, buccal, and palatal cusp tips were used. This was followed by conducting an automated iteration process to orient the images according to the best‐fit alignment. Subsequently, the root mean square (RMS) in μm was calculated to quantify the deviation between the two images by measuring average deviation between numerous corresponding points at the two images according to the following equation:

$$\mathrm{RMS}=\sqrt{\frac{\sum {\left(R_{i}-\;C_{i}\right)}^{2}\;}{n}}$$

where *R*_*i*_ is a point of the first image, *C*_*i*_ is the corresponding point of the second image, and *n* is the total number of points.

The less the RMS value, the greater the trueness and precision. The trueness was supplemented by qualitative analysis, where a color‐coded map was produced for every image at the PI level to determine the pattern of deviation of each preparation by every scanning unit. According to the software, accurate fitting areas were colored in green. Warm colors on the map implied positive errors where the IOS image is dimensionally larger than the original virtual image, whereas cool colors indicated negative errors where the IOS image was smaller than the original virtual image.

The mean and SD of trueness and precision of PI and SI levels were calculated for each preparation and IOS system. The Shapiro Wilk test was applied to confirm the normality of the data. The two‐way ANOVA test was applied for the PI and SI levels, followed by post hoc tests for intragroup comparison. The statistical tests were conducted through the SPSS software package (SPSS for Windows, v23; SPSS Inc, Chicago, Illinois), and the level of significance was set at 0.05.

## 3 RESULTS

### 3.1 Trueness

**FIGURE 3:**
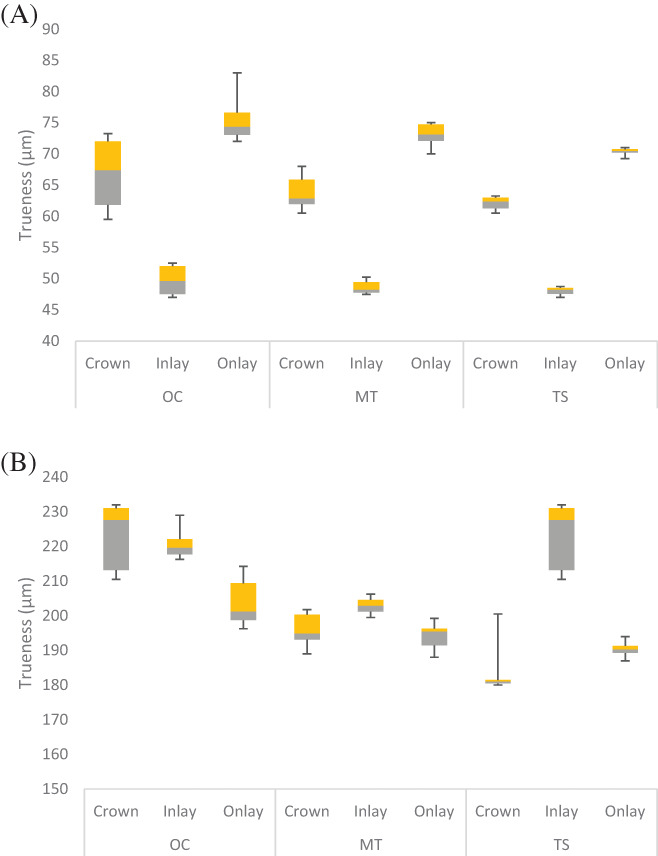
Box and Whisker plot summarizing the trueness (μm) of the different preparations for each IOS system at the different levels (*n* = 10). (A) PI level. (B) SI level

At the PI level (Table 2), all the IOS systems had a similar trueness pattern for all preparations, where the inlay had the best trueness accuracy, followed by crown and onlay (*p* < 0.001), respectively (Figure 3A). For all IOS systems, the pairwise comparison revealed significant differences among the different preparations (*p* < 0.001). At the SI level, the trueness had general similarities in values among the different preparations and IOS systems (Figure 3B). For OC, a significant difference was found between the different preparations (*p* < 0.001), where the onlay had fewer trueness errors than crown (*p* < 0.001), and inlay (*p* < 0.001). The difference between crown and inlay was insignificant (*p* = 0.69). The MT showed significantly superior trueness for onlay, followed by crown and inlay (*p* < 0.001). However, this difference was insignificant between crown and onlay (*p* = 0.45). The TS had the most inferior outcome for inlay followed by onlay and crown (*p* < 0.001), respectively. The pairwise comparison among the different preparations revealed significant differences for each comparison for TS (*p* < 0.05).

**TABLE 2:** Trueness accuracy at the PI and SI levels in μm (*n* = 10)

	PI level								
	OC			MT			TS		
	Crown	Inlay	Onlay	Crown	Inlay	Onlay	Crown	Inlay	Onlay
Mean (μm)	66.7^A,a,^*	49.7^B,a,^*	75.3^C,a^*	63.8^A,a,b,^*	48.6^B,a,b,^*	73.2^C,a,^*	62.2^A,b,^*	48.1^B,b,^*	70.4^C,b,^*
Standard deviation (μm)	5.1	2.1	3.2	2.6	1.0	1.6	0.9	0.6	0.5
Median (μm)	67.4	49.6	74.4	62.9	48.3	73.1	62.4	48.3	70.5
Maximum (μm)	73.3	52.5	83.0	68.0	50.3	75.0	63.3	48.8	71.0
Minimum (μm)	59.5	47.0	72.0	60.5	47.5	70.0	60.5	47.0	69.25

	SI level								
	OC			MT			TS		
	Crown	Inlay	Onlay	Crown	Inlay	Onlay	Crown	Inlay	Onlay
Mean (μm)	223.0^A,a,^*	220.5^A,a,^*	203.1^B,a,^*	196.1^A,b,^*	202.9^A,a,^*	194.2^A,b,^*	182.9^A,c,^*	223.0^B,b,^*	190.4^C,b,^*
Standard deviation (μm)	8.9	3.9	5.9	4.3	2.2	3.6	6.2	8.9	1.8
Median (μm)	227.6	219.6	201.3	194.9	202.9	195.5	181.0	227.6	190.3
Maximum (μm)	232.0	229.0	214.3	201.8	206.3	199.3	200.5	232.0	194.0
Minimum (μm)	210.5	216.3	196.3	189.0	199.5	188.0	180.0	210.5	187.0

Abbreviations: MT, Medit i500; OC, Cerec Omnicam; TS, Trios 3.

*Different superscript letters next to the mean values indicate statistical difference between groups (multiple pairwise comparison) (*p* < 0.05). Uppercase letters indicate the comparison between different preparations within the same IOS system. Lower case letters indicate the comparison between the different IOS systems for each preparation.

Comparing the trueness of different scanners revealed significant differences among the IOS systems for every preparation at the PI and SI levels (*p* < 0.001). At the PI level, there was no difference between OC and MT for the inlay (*p* = 0.62), crown (*p* = 0.15), and onlay (*p* = 0.07). The MT and TS were comparable for inlay (*p* = 0.71) and crown (*p* = 0.54). The TS had superior trueness to MT for onlay (*p* = 0.02). The TS was consistently more superior than OC for all the preparations (*p* < 0.05). At the SI level, the crown had the best trueness for TS followed by MT and OC, respectively (*p* < 0.05). For inlay, MT showed the most superior trueness followed by OC then TS. However, no significant difference was found between OC and MT (*p* = 0.62). TS had the best onlay trueness followed by MT and OC at the SI level. No significant difference between MT and TS was observed for onlay (*p* = 0.11) at the SI level.

### 3.2 Precision

**FIGURE 4:**
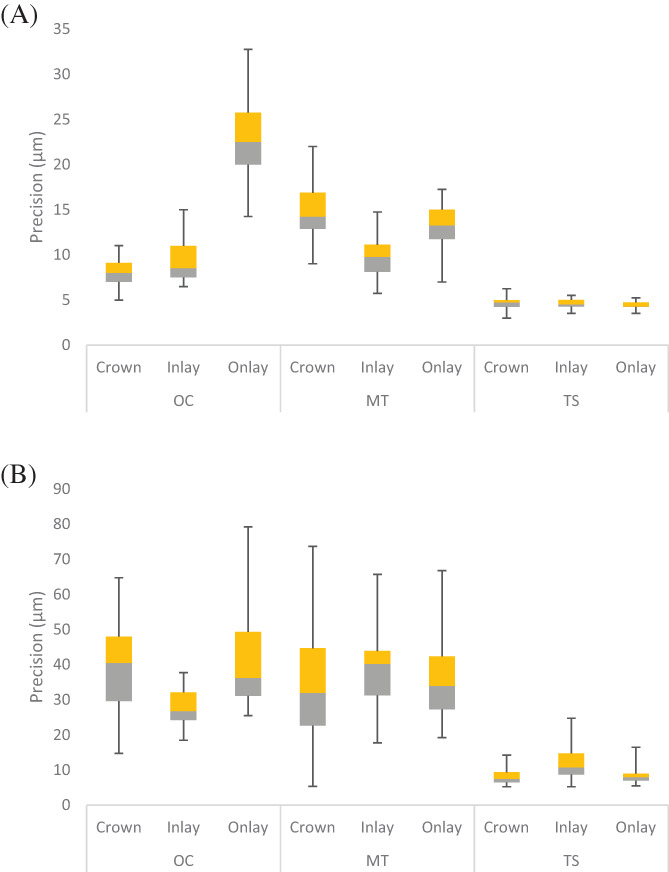
Box and Whisker plot summarizing the precision (μm) of the different preparations for each IOS system at the different levels (*n* = 45). (A) PI level. (B) SI level

Regardless of the level of comparison, TS was more precise than all scanners (Table 3). At the PI level (Figure 4A), the OC revealed a superior outcome of the crown than inlay and onlay, respectively (*p* < 0.001). However, the difference between crown and inlay was minimal (*p* = 0.11). For the MT, the inlay had a significantly superior outcome followed by onlay and crown. The difference among them was significant for all the comparisons (*p* < 0.05). TS showed no significant difference among the different preparations (*p* = 0.08). At the SI level (Figure 4B), OC had significantly superior precision for inlay (*p* < 0.001) than crown and onlay, which were very similar (*p* = 0.95). On the contrary, TS had inferior precision for inlay (*p* < 0.001) than crown and onlay, which had similar precision (*p* = 0.97). The MT showed similar SI precision for the different preparations (*p* = 0.56).

**TABLE 3:** Precision accuracy at the PI and SI levels in μm (*n* = 45)

	PI level								
	OC			MT			TS		
	Crown	Inlay	Onlay	Crown	Inlay	Onlay	Crown	Inlay	Onlay
Mean (μm)	8.1^A,a,^*	9.3^A,a,^*	23.0^B,a,^*	14.9^A,b,^*	9.9^B,a,^*	13.2^C,b,^*	4.6^A,c,^*	4.6^A,b,^*	4.4^A,c,^*
Standard deviation (μm)	1.4	2.2	4.3	3.4	2.2	2.5	0.7	0.5	0.4
Median (μm)	8.0	8.5	22.5	14.3	9.8	13.3	4.8	4.5	4.3
Maximum (μm)	11.0	15.0	32.8	22.0	14.8	17.3	6.3	5.5	5.3
Minimum (μm)	5.0	6.5	14.3	9.0	5.8	7.0	3.0	3.5	3.5

	SI level								
	OC			MT			TS		
	Crown	Inlay	Onlay	Crown	Inlay	Onlay	Crown	Inlay	Onlay
Mean (μm)	39.6^A,a,^*	27.5^B,a,^*	40.3^A,a,^*	36.1^A,a,^*	38.5^A,b,^*	35.7^A,a,^*	8.3^A,b,^*	12.7^B,c,^*	8.4^A,b,^*
Standard deviation (μm)	12.4	4.7	12.5	17.2	11.0	10.8	2.5	5.4	2.4
Median (μm)	40.5	26.8	36.3	32.0	40.3	34.0	7.5	10.8	8.0
Maximum (μm)	64.8	37.8	79.3	73.7	65.8	66.8	14.3	24.8	16.5
Minimum (μm)	14.8	18.5	25.5	5.3	17.8	19.3	5.3	5.3	5.5

Abbreviations: MT, Medit i500; OC, Cerec Omnicam; TS, Trios 3.

*Different superscript letters next to the mean values indicate statistical difference between groups (multiple pairwise comparison) (*p* < 0.05). Uppercase letters indicate the comparison between different preparations within the same IOS system. Lower case letters indicate the comparison between the different IOS systems for each preparation.

Comparing the precision of the different scanners for each preparation indicated significant differences among the scanners at the PI and SI levels. For the crown, TS had the most superior PI precision followed by OC, which had significantly superior precision to MT (*p* < 0.001). Similarly, TS showed the most superior SI crown precision (*p* < 0.001), followed by MT and OC which were similar (*p* = 0.39). For PI and SI levels of inlay, the differences among the IOS systems were significant (*p* < 0.001), and the superior outcome was for TS followed by OC and MT. All the comparisons were significant, except at the PI level between OC and MT (*p* = 0.33). For PI and SI levels of onlay, the best outcome was for TS, followed by MT and OC. The difference among all the comparisons were significant (*p* < 0.001), except at the SI level between OC and MT (*p* = 0.07).

### 3.3 Qualitative results

**FIGURE 5:**
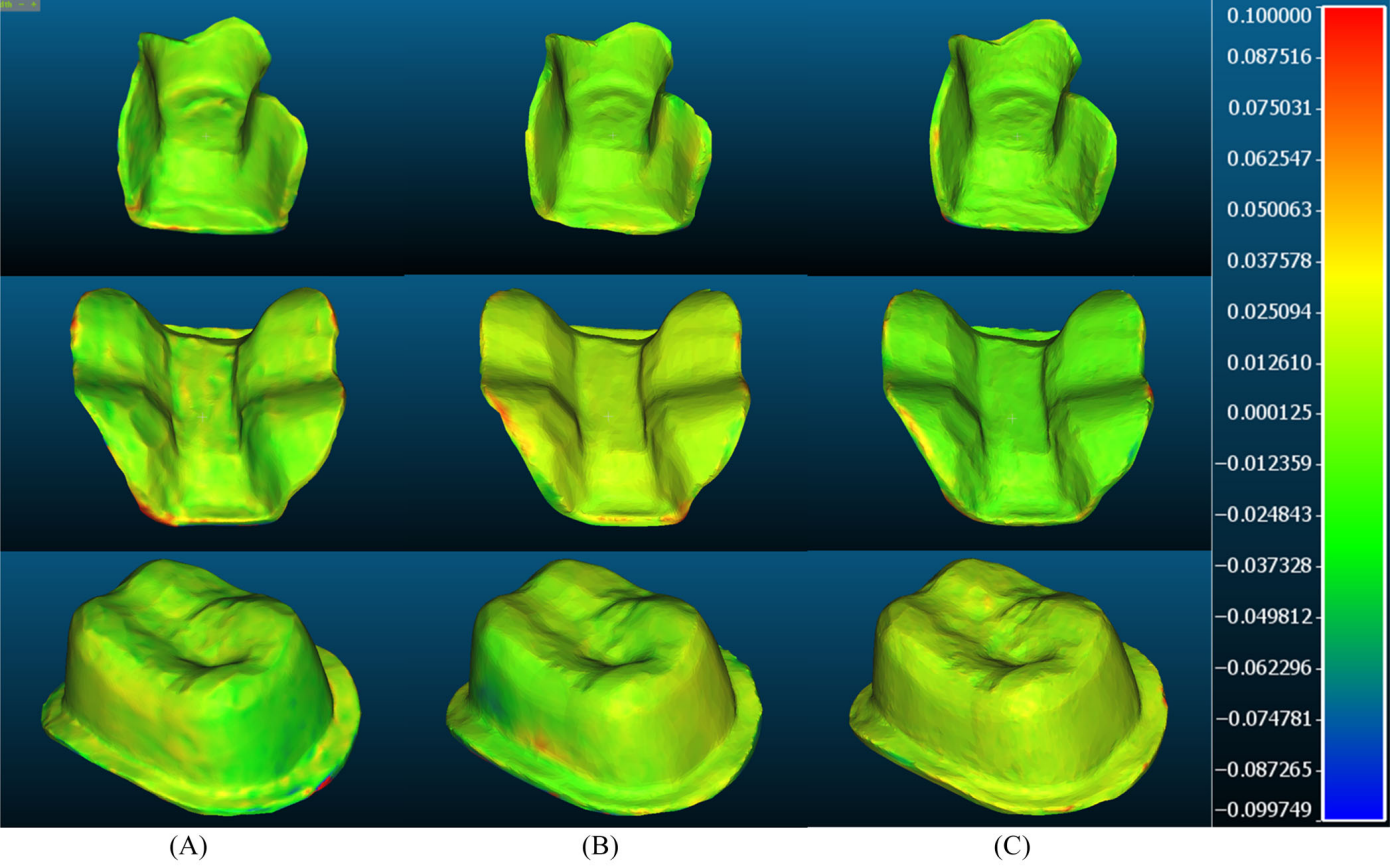
Examples of qualitative images of the different preparations. The inlay preparation showed general superiority than other preparations, while the onlay preparation was associated with greatest errors regardless of IOS systems. The proximal surfaces were more prone to errors than other surfaces. (A) OC. (B) MT. (C) TS

The qualitative images showed a general superiority of the inlay followed by crown and onlay, respectively (Figure 5). The inlay images showed relative accuracy of capturing all the surfaces and the margins. The inlay and onlay proximal margins tended to suffer from some errors that were more obvious for OC, in the form of positive rounding and loss of definition, compared with the more defined images of MT and TS. In addition, while the area beyond the proximal margin is less critical, there were clear signs of errors in the OC images in comparison with the other IOS scanners. Onlay images were distinguished with the greatest distribution of surface errors for all IOS systems. In addition to the proximal surface, the occlusal margins showed signs of predominantly positive errors.

The crowns generally showed accurate recordings of the occlusal and axial surfaces, and the margins tended to be more accurate than for inlays and onlays. The proximal axial surfaces showed signs of positive errors. The margins showed the greatest errors and roundness that were noticeable for all IOS systems.

## 4 DISCUSSION

This study revealed that the type of preparation and the IOS system can influence the accuracy of digital impression. Specifically, the preparation type had a clear impact on trueness at the PI level, while the scanner affected the precision at the PI and SI levels. Therefore, the hypotheses that there is no effect of the different preparation type on the accuracy of IOS, and that there is no difference between the different IOS systems were rejected. The differences in trueness of the different preparations at the PI level can be attributed to the geometry of the preparation and its implication on the scanning process, The accuracy of the light scanner is enhanced by scanning the accessible surfaces at the perpendicular direction, while surfaces captured at an angle are likely to suffer from errors in the form of missing data or rounding of line angles.^2^, ^3^, ^4^, ^5^, ^6^, ^7^, ^9^ Thus, the superiority of inlay preparation at the PI level can be related to the accessibility and simplicity of the preparation surfaces, which can further translate into less movement of the scanner and quicker scanning.^10^, ^14^ On the other hand, the onlay preparation is distinguished with the more complex and corrugated morphology, and larger preparation extending to the mesial and distal aspects of the tooth, which requires more scanning time and movement of the scanning camera to capture all the surfaces. With increased scanning duration and movement, a greater accumulation of registration errors will likely cause increased deterioration of the final accuracy.^14^ The presence of irregular surfaces that followed cuspal anatomy may further challenge the scanning procedure by exposing the surfaces to scanning rays at multiple angles. This was corroborated by several studies that showed complex and irregular surfaces were prone to greater errors when scanned intraorally.^4^, ^5^, ^9^, ^14^, ^15^, ^16^, ^17^, ^18^, ^19^, ^20^ On the other hand, the crown preparation may have the slight advantage over the onlay preparation due to the even supragingival margin placement across the tooth,^4^, ^9^, ^13^, ^15^ and the rounded line angles which further simplify the scanning process.^21^ In addition, the proximal margins of the crown preparation were more accessible for scanning with the absence of proximal box walls.^3^ Several studies recommended a distance of at least 0.5 mm from the preparation margin to the adjacent tooth for acceptable scanning, which was easily provided with the crown preparation of the study.^2^, ^3^, ^4^, ^7^ Nevertheless, all preparations showed an accuracy of less than 100 μm at the PI level, which is considered clinically acceptable for long‐term prognosis.^2^, ^3^, ^9^, ^15^

The qualitative evaluation clearly indicated that not all surfaces were captured at similar accuracy. Irrespective of the preparation type, there is a tendency for all preparations to exhibit inferior outcomes at the proximal areas, which was observed by numerous earlier studies.^1^, ^2^, ^3^, ^22^, ^23^ This is thought to be caused by the limited visibility of the proximal regions to scanning and angled scanning orientation.^1^, ^5^ In particular, the presence of adjacent teeth prevents perpendicular scanning of the proximal regions, which mandates orienting the camera parallel to the proximal axial surfaces that can further lead to missing some areas and relying on the software to estimate the missing regions.^3^, ^5^ The crown preparations showed better proximal margin quality, which can be attributed to the more accessible proximal regions. On the contrary, the proximal surfaces of inlays and onlays showed more noticeable errors qualitatively, which was most likely accentuated by the presence of proximal boxes and walls that are closer to the adjacent teeth. Thus, it is reasonable to consider deliberate flaring of the proximal boxes to create clearer separation from the adjacent tooth, and to facilitate the scanning process and accessing the preparation margin.^2^, ^7^ In addition, scanning errors were observed in the cervical region below the preparation margin. While this region has a minimal impact on the fit of the restoration, it may influence the virtual determination of restoration margin and contour. In the present study, the local proximal deviation appears to be 2–3 times the deviation of the rest of the preparation surface, and can reach a magnitude of 200 μm. The errors of the scanned images are predominantly positive (larger than the actual tooth) which may translate to an underextended restoration margin, thinner restoration, and thicker cement layer.^1^, ^2^, ^24^ While positive deviations can have less immediate clinical consequences than negative deviations, it may eventually encourage loss of margin integrity, cement dissolution, and sensitivity.^1^

The SI level errors were approximately 4–5 times the errors at PI level for all preparations, which can be attributed to the span of evaluation. For example, despite the superiority of inlay preparation over other preparations at PI level, the evaluation at the SI level revealed inferior trueness than other preparations. The increased span of evaluation, along with the adjacent teeth, eliminated the advantages of the simple inlay preparation scanning. Specifically, the inlay preparation with the proximal box and no cuspal reduction may create shadowing and reduce accurate recording of the adjacent tooth.^2^, ^3^, ^5^, ^8^ In addition to the prepared tooth, the SI evaluation included axial undercuts, proximal surfaces, and corrugated occlusal anatomy of adjacent teeth.^1^, ^2^, ^3^, ^5^, ^7^, ^8^, ^21^ Some surfaces from the adjacent teeth will inevitably be missed and patched by the software, which contributes to the increased error.^2^, ^3^ Moreover, the differences between PI and SI levels could be due to research methodology limitations. For example, at the PI level, the manual alignment points were placed at close proximity in comparison to the points at the SI level, which can translate to more reliable best‐fit alignment. Due to the considerable accuracy difference between PI and SI levels, studies on the accuracy of IOS should differentiate between the outcome of preparation surface scanning or segment scanning. As the deterioration is expected to occur at the adjacent teeth and prepared tooth area beyond the preparation margin, it can be argued that these areas are of minimal significance to the fit of the definitive restoration. Nevertheless, the clinical significance of the inferior recording of adjacent teeth surfaces is yet to be evaluated by future studies.

The comparison among the different IOS systems indicated that the TS was clearly more precise than MT and OC, respectively. This superiority was observed by several earlier studies,^11^, ^12^, ^13^ and was attributed to the confocal microscopy scanning mechanism of TS, which is assumed to be a more accurate triangulation‐based mechanism of MT and OC.^11^, ^12^, ^13^ However, this is negated by other studies that did not show similar accuracy patterns.^1^, ^2^, ^3^, ^24^ Nevertheless, the TS may have the advantages of being a relatively newer system than the other IOS systems, which may have contributed to easier scanning, more user‐friendliness, superior hardware, scanning time, and image resolution. More importantly, as the differences among the scanners are only affecting precision, which is a measure of repeatability, it can be argued that the actual difference among them is minimal, and the repeatability advantage of TS may not necessarily translate to more accurate restorations.^2^, ^3^ In accordance with the present study, earlier studies indicated a lack of correlation between trueness and precision, and IOS systems tend to show superior precision values than trueness values.^1^, ^2^, ^3^, ^13^, ^24^ Thus, the differences in precision could be significant from the statistical perspective, with minimal clinical impact. More importantly, as the trueness pattern is similar for the different IOS systems for each preparation type, the influence of each preparation on the accuracy can be more relevant clinically.

The present study is limited in evaluating the accuracy of IOS at the image acquisition stage only. While the errors observed in the present study are of minimal magnitude, they will still contribute to the accumulated errors of the definitive restoration.^1^, ^2^, ^3^, ^15^ Additional errors are inevitable from the subsequent steps, such as virtual margin determination, virtual restoration design, and restoration production. Nevertheless, the available studies on IOS‐based restorations showed that IOS can produce restorations with margins less than 200 μm, which is considered within the acceptable clinical range.^1^, ^4^, ^15^, ^24^ However, the laboratory nature of the present study prevents translating its results to a clinical set‐up. Clinically, IOS use is further challenged with variations in tooth preparation, limited mouth opening, less accessible tooth locations, presence of saliva, presence of tongue and intraoral tissues, and possible patient movement. As the study involved scanning only a single unopposed arch, the laboratory conditions may have permitted greater freedom for scanning and have optimized the IOS accuracy.^2^, ^3^ Therefore, while it is tempting to suggest that inlays are superior to crowns and onlays through the IOS protocol, this cannot be extrapolated clinically. Other studies have reported dissimilar outcomes where predominantly intracoronal preparations exhibited inferior outcomes to extracoronal preparations.^1^, ^13^, ^15^ Features such as preparation depth, margin location, convergence angle, and proximal area design are anticipated to further affect the IOS quality regardless of the preparation type.^2^, ^4^, ^9^, ^13^

## 5 CONCLUSION

Within the limitations of the present study, it can be concluded that the smaller and less complex preparations have greater IOS accuracy than larger and more complex preparations. This effect was found for trueness accuracy at the PI level. As the proximal areas are more affected regardless of the preparation, a more accessible proximal area for scanning is desirable. While different IOS systems may exhibit different precision, the trueness accuracy appears to be minimally affected by the different IOS systems.

## FUNDING INFORMATION

This research did not receive any specific grant from funding agencies in the public, commercial, or not‐for‐profit sectors.

## Data Availability

The data that support the findings of this study are available from the corresponding author upon reasonable request.
